# Dealing with Target Uncertainty in a Reaching Control Interface

**DOI:** 10.1371/journal.pone.0086811

**Published:** 2014-01-28

**Authors:** Elaine A. Corbett, Konrad P. Körding, Eric J. Perreault

**Affiliations:** 1 Sensory Motor Performance Program, Rehabilitation Institute of Chicago, Chicago, Illinois, United States of America; 2 Department of Physical Medicine and Rehabilitation, Northwestern University, Chicago, Illinois, United States of America; 3 Department of Physiology, Northwestern University, Chicago, Illinois, United States of America; 4 Department of Biomedical Engineering, Northwestern University, Chicago, Illinois, United States of America; Bielefeld University, Germany

## Abstract

Prosthetic devices need to be controlled by their users, typically using physiological signals. People tend to look at objects before reaching for them and we have shown that combining eye movements with other continuous physiological signal sources enhances control. This approach suffers when subjects also look at non-targets, a problem we addressed with a probabilistic mixture over targets where subject gaze information is used to identify target candidates. However, this approach would be ineffective if a user wanted to move towards targets that have not been foveated. Here we evaluated how the accuracy of prior target information influenced decoding accuracy, as the availability of neural control signals was varied. We also considered a mixture model where we assumed that the target may be foveated or, alternatively, that the target may not be foveated. We tested the accuracy of the models at decoding natural reaching data, and also in a closed-loop robot-assisted reaching task. The mixture model worked well in the face of high target uncertainty. Furthermore, errors due to inaccurate target information were reduced by including a generic model that relied on neural signals only.

## Introduction

People will almost always look at an object before reaching for it [1], providing us with a rich source of information about their intended arm movements. Such a means of decoding intent may be useful for a range of user interface applications [2], including the restoration of communication or movement to people whose arms have been paralyzed. As eye-tracking systems become less expensive this signal source is gaining attention as a viable, non-invasive tool for such rehabilitation applications [3]. However, gaze may be a problematic input signal when used alone as it can be difficult to determine which saccades are intended as control signals, and it is very challenging to control eye movements precisely for extended periods of time. In particular, if the purpose of the interface is to restore movement with a neuroprosthesis it is vital that saccades away from an intended reach target do not generate unintentional commands. Furthermore, the interface would be unlikely to succeed if it restricted the user’s ability to look around their workspace. Gaze may be an extremely useful control signal, but the user interface must be able to safely deal with the associated uncertainty.

For the severely impaired, the set of physiological signals under voluntary control that can be used in a rehabilitative interface may be extremely limited. In such cases, it often makes sense to combine control information from disparate signal sources [4], [5]. For example, Batista et al. found that they could improve target selection performance in a brain-computer interface by monitoring eye movements [6]. A number of groups have improved trajectory decoding by taking advantage of the directional nature of reaching [7]–[9], by combining information about the reach target with neural trajectory control signals. Such target information may come from recordings in the dorsal pre-motor cortex [10], [11], and sometimes with prior knowledge of the distribution of potential targets [12]. Following this logic, we used target estimates from gaze to enhance our model of the reach trajectory when decoding reaching movements with electromyograms (EMGs) [13]. We evaluated this interface with a robot-assisted reaching task, as a proxy for a neuroprosthesis, finding that the incorporation of gaze improved control and reduced the burden on the user [14].

As people may saccade to other locations in addition to the reach target, it is necessary to account for uncertainty in the target estimates. This was accomplished in our algorithm by using a mixture model initiated with a probabilistic distribution of potential targets obtained from the gaze in the one-second period before the reach [14]. However, our experiments were performed under controlled conditions with reasonably attentive subjects. While our decoder was designed to account for multiple potential targets, its performance under highly uncertain conditions had not been directly evaluated. Furthermore, the algorithm made the assumption that the target would be foveated in the period preceding the reach. While this was a generally safe assumption, in a worst-case scenario in which the target was never foveated, incorrect target information could cause unwanted movements. A user may initiate a reach unintentionally or be distracted and look away from the target. Additionally, calibration issues with the eye tracker could result in inaccurate target estimates. These may be rare occurrences, but due to the potentially high cost of an error they are important to consider. A safe and reliable neuroprosthesis interface would need to deal with these situations gracefully.

Decoding performance in the face of target uncertainty will depend on the amount of neural or other continuous physiological signals being incorporated, as those signals inform the decoder about the probability of each potential target. The mixture model considers a different trajectory for each of the potential targets, and weights them probabilistically. As the continuous signals are integrated over the course of the reach, their likelihood is evaluated for each of the trajectories and used to assign the weights. In [13] we evaluated the decoding of natural human reaching, combining target estimates from gaze with different sets of EMGs in an attempt to simulate the control signals that might be available at different levels of spinal cord injury (SCI). While incorporating gaze clearly reduced the reliance on the EMG, the decoding still suffered somewhat when the number of available EMG signals was most limited. In our closed-loop evaluations, in able-bodied subjects performing a robot-assisted reaching task, we used EMGs typically available at the fourth and fifth cervical levels (C4 and C5) and there was no difference in accuracy between the two conditions [14]. However, performance appeared to be more reliant on the accuracy of the gaze data in the C4 case. The gaze produced by the subjects during simulated C5-level control was less accurate, presumably because they could compensate for errors using the additional EMG signals that were included in the interface. This indicated that it may be more difficult to compensate for target uncertainty in the C4 case, with fewer EMGs, and its effect on reach accuracy was unknown. Without a rich set of neural data, it may be difficult for the decoder to select the correct target in uncertain conditions.

Here we considered what happened as the quality of our target estimates declined. We tested the decoding of natural reaching data at the various simulated injury levels while replacing the gaze with simulated noisy target estimates, systematically evaluating the performance of the mixture model and its dependence on the available neural signals. We also proposed an extension to the algorithm that accounted for the worst-case scenario where the target was not foveated by including a generic model with no target information into our mixture model, giving more control to the user’s neural signals when they indicated that none of the target estimates were likely to be correct. Finally, we evaluated the original and modified mixture models in closed-loop robotic control of the arm, again with simulated noisy target estimates. C4, the most impaired simulated injury level, was used for the closed-loop evaluation because it was at this level that the inclusion of target information produced the most substantial performance gains. Furthermore, it provided the most challenging test for the mixture models where the EMG was most limited. We found that subjects could adapt to the mixture models and deal well with target uncertainty, and the algorithm modification helped substantially when the target information was inaccurate. Portions of this work were presented previously in a conference publication [15].

## Methods

In previous work we developed and tested a mixture of targets model that incorporated gaze with EMG in a reach decoder [13]. Here we simulated uncertain target information to evaluate how it would perform in the presence of “noisy gaze” when subjects became distracted or foveated locations in addition to the target. We also proposed an extension to the mixture model to mitigate the worst-case scenario when the target information is inaccurate. These approaches were tested in both an offline decoding setting and in closed-loop control of robot-assisted reaching. The original and extended mixture models were compared, with different levels of target uncertainty, to a decoder driven by EMG alone. The algorithms that are common to both experiments were described first. The three decoders were then compared with varying quantities of EMG data, simulating different levels of SCI, in an offline analysis using the natural reaching data from [13]. They were then tested at the most impaired simulated injury level in closed-loop control using a robot-assisted reaching task.

### Ethics Statement

All subjects provided informed written consent to the experimental protocol, which was approved by Northwestern University's Institutional Review Board.

### Decoding Algorithms

With no prior knowledge of the target location, our best prediction of the reach kinematics should come from a decoder driven by neural data alone. For this case we used the generic Kalman filter (KF) [16] framework where an observation model, defining the mapping from the neural data to the kinematics, is combined with a trajectory model, describing the evolution of the kinematics over the course of the reach. We defined a trajectory model where the kinematics evolved linearly over time while integrating Gaussian noise:
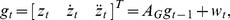
(1)where *z_t_* represented the hand or arm position vector at time *t*, *g_t_* was the state vector consisting of *z_t_* and its first and second derivatives, and *A_G_* was the state transition matrix for the generic trajectory model. The process noise, *w_t_,* had a zero-mean, Gaussian probability distribution, p(*w*) ∼ *N*(0,*Q*), where *Q* was the state covariance matrix.

We used a linear Gaussian observation model to map features of the EMG to the reach kinematics:
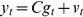
(2)where *y_t_* was the EMG feature vector at time *t*, *v_t_* was Gaussian noise with p(*v*) ∼ *N*(0,*R*), and *R* was the observation covariance matrix. We chose the root-mean-squared (RMS) value (a measure of the magnitude) and number of zero crossings (a frequency measure) in the sample time-window of EMG as features, and square-root-transformed them for more Gaussian-like distributions.

In the KF recursion at each time-step, the *a priori* estimates of the current state and covariance were predicted from their *a posteriori* estimates at the previous time-step through the trajectory model. These *a priori* predictions were then updated using the current time-step's observation, and the *a posteriori* estimate was found as an optimal combination of the two models' predictions.

To create a Kalman filter with target information (KFT), we employed a directional trajectory model by adding the target position into the state vector, assuming a linear time-invariant effect on the kinematics:
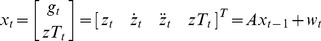
(3)where z*T_t_* was the target position vector, with dimensionality less than or equal to that of z*_t_* and *A* was the state transition matrix for the directional KFT model. The same observation model from equation 2 was used to evaluate the KFT, with *x_t_* in place of *g_t_*.

When accounting for uncertain target information, as when target estimates were based on eye movements, the KFT was evaluated for multiple potential targets. We obtained a prior distribution for the possible targets, *P(zT_t_)*, which in our previous work we obtained from the subjects' gaze. As the neural data were integrated over the course of the reach (*y_1….t_*), it informed us about the likelihood of each of the possible resultant trajectories. A probabilistic mixture model (mKFT) over each of the *N* potential targets *zT* provided our final state estimate:
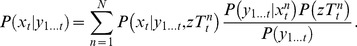
(4)


The KF recursion was performed for each potential target, *zT^n^*, and the predicted state, *P(x_t_ | y_1….t_)*, was a weighted sum of the state estimates from each model, *P(x_t_ | y_1….t_,zT_t_^n^)*, where the weights were proportional to the prior for the associated target, *P(zT_t_^n^)* and the likelihood of the trajectory given that target *P(y_1….t_ | x_t_^n^)*. *P(y_1….t_)* was independent of the target and therefore was used as a scaling factor to ensure that the weights summed to 1. Thus, the weights are initialized to the prior probabilities assigned to them, but as the EMG information comes in over the course of the reach the likelihoods of the EMG for the different trajectory models should diverge, causing the weight for the more probable model to dominate.

Here we wanted to modify the algorithm to recover as safely and gracefully as possible if the target information failed. If our information about the reach target was inaccurate, the best we could hope for would be that, rather than following a trajectory to an incorrect target, the reach could be guided with the subject’s neural control signals alone. With this goal in mind, we added the generic KF component into the mixture model (mKFT+KF):
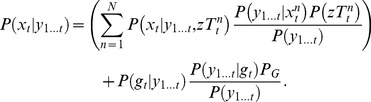
(5)


If for each reach the generic model was initiated with a low prior probability, *P_G_*, it would only come into play if the neural data indicated that none of the other potential trajectories were probable. As the EMG information was integrated over the reach, if its likelihood for the generic model (*P(y_1….t_ | g_t_)*) was high relative to the directional trajectory models then the weight of the generic model would dominate; otherwise, the weight for the generic model should remain close to zero.

### Offline Evaluation of Natural Reach Decoding

We first compared the offline decoding accuracy of the generic KF (EMG alone) to that of the two mixture models, with varying amounts of target uncertainty, using unconstrained 3D reaching data over a wide range of dynamics from [13]. We used three sets of EMGs recorded from able-bodied human subjects, simulating C6, C5 and C4-level SCIs. While data from able-bodied reaching are not necessarily representative of real neuroprosthesis use, this analysis allowed us to study the effect of the quantity of neural data on the two mixture models as they dealt with target uncertainty. The data collection is briefly summarized below, followed by a description of the analyses.

#### Data collection

**Figure 1:**
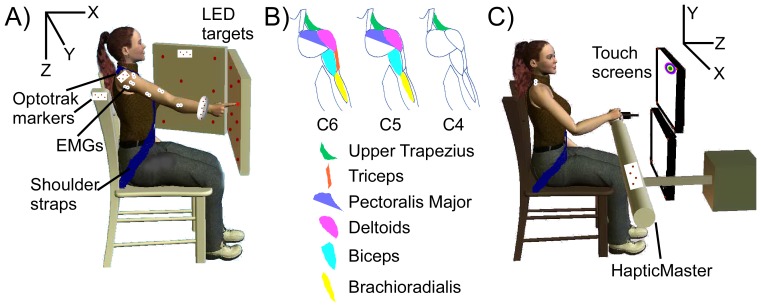
Experimental setups A) Natural reaching data; B) Muscles recorded to simulate levels of SCI; C) Closed-loop experiment.

We recorded arm kinematics and EMGs from five able-bodied subjects performing reaches towards 16 LED targets, located on two planes positioned such that all of the targets were just reachable (figure 1a). Each subject performed between 450 and 500 reaches at varying speeds, as might occur in everyday life, while comfortably seated and restrained with lap and shoulder straps. EMG signals were recorded from the ipsilateral brachioradialis, biceps, long and lateral heads of the triceps, pectoralis major (clavicular head), posterior, middle and anterior heads of the deltoid and upper trapezius. The EMG signals were amplified and band-pass filtered between 10 and 1,000 Hz using a Bortec AMT-8 (Bortec Biomedical Ltd, Canada), anti-aliased filtered using 5th order Bessel filters with a cut-off frequency of 500 Hz, and sampled at 2,400 Hz. Hand, wrist, and shoulder positions were tracked at 60 Hz using an Optotrak motion analysis system (Northern Digital Inc, Canada).

#### Analysis

For each subject, 100 reaches were randomly selected for testing while the remainder were used to train the models. In the state vector we used joint angles (3 shoulder, 2 elbow) and finger position, velocity and acceleration. Joint angles were calculated from the shoulder and wrist marker data using digitized bony landmarks which defined a coordinate system for the upper limb as detailed by Wu et al. [17]. We performed the analysis with three different sets of EMGs, simulating the residual muscle activity typical at different levels of SCI. To simulate a C4-level injury we used just the upper trapezius; for C5 we added the three heads of the deltoid, the biceps, the pectoralis major and the brachioradialis; for C6 we also included the two heads of the triceps, which may have residual activity in this population (figure 1b). As we performed decoding at 60 Hz, the EMG features were extracted from the corresponding 16.7 ms windows to be used as observations of the state at each time-step. We estimated the decoder parameters from the EMG and arm kinematic data using least squares regression. In the case of the directional trajectory model estimation (KFT), the final position of the finger at the end of the reach was inserted into the state vector, representing the target.

We evaluated the decoding approaches by comparing their predictions of the finger position in the test reaches. To evaluate performance with different levels of target uncertainty we simulated the potential targets. Specifically, for the mKFT, we initiated each reach with two potential targets – the actual finger position at the end of the reach and a randomly selected position from the set of potential targets. We then varied the prior for the random target between 0 (perfect target information) and 1 (completely random target information). The mKFT+KF was tested with the same simulated targets. The prior for the generic model was selected to be *P_G_* = 0.1 and the non-zero priors for the remaining targets were reduced each by 0.05 or 0.1 if there was only one target represented in the mixture.

For the mKFT+KF we quantified a histogram for the value of the weight assigned to the generic model for each level of target uncertainty (prior for the random target). Algorithm accuracy was quantified using the multiple R^2^ [18], which is a measure of accuracy that incorporates the entire reaching movement. Intuitively, the multiple R^2^ evaluates the accuracy across all three dimensions, weighing each dimension in proportion to its variance. However, because all targets were limited to two planes, a substantial component of this R^2^ was related to movement common to all reaches. Hence, we also quantified the error at the final time of the reach using the target variance accounted for (VAF), by scaling the squared error at that time by the variance in the LED target positions:
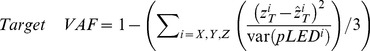
(6)where *T* is the final time of the reach, *pLED* are the LED locations in space, and *i* indexes the dimensions X, Y and Z. The conditions were compared using an ANOVA where the *simulated injury level* and the combination of *algorithm* and the *prior probability assigned to the random target* were included as fixed effects, and *subject* was a random effect. A Tukey post-hoc test was performed and all statistical comparisons used α = 0.05.

### Closed-loop Evaluation

A robotic system to assist reaching was used to test the decoders in closed-loop control. The robotic system accurately positioned the arm based on the decoded kinematics, thereby isolating performance issues relevant to decoders and signal sources and providing a wider subject population on which to evaluate the approaches. By moving the subject's arm along with the decoded reaches, the subjects were provided with a more realistic feedback consistent with neuroprosthesis use than would be available with a virtual interface. Furthermore, it ensured that the EMG signals were consistent with the movement of the arm.

The simulated C4-level injury was tested in closed-loop control because it had demonstrated the greatest performance increase from the inclusion of target information in our previous work. Also, the offline results (see below) indicated that the system would perform well in the face of target uncertainty with sufficient EMG. As there was only a single EMG available at C4 to inform us about the probability of the various mixture components, the effects of the target priors could be expected to be greater than at other simulated injury levels. We did not know how well subjects would be able to manipulate this interface with uncertain target priors; in our previous experiments the accuracy of the mKFT had appeared to be driven almost entirely by the precision of the target estimates from gaze. For more detailed descriptions of the experimental paradigm see [14].

#### Subjects and experimental setup

Five able-bodied, right-arm dominant subjects took part in this experiment. Four of the five subjects had previously participated in similar experiments [14]. Each subject gripped a 3 degrees of freedom HapticMaster robot (Moog FCS, the Netherlands), mounted at 90° to the wall, which moved the subject’s right hand throughout a reaching workspace (figure 1c). The robot was stiff (20,000 N/m) and could not be manipulated by more than a few millimeters by voluntary forces at the hand. A spring-loaded stylus attached to the robot handle allowed for soft contact with targets that were displayed on two 37 cm×30 cm touch-screen monitors (Planar PT19, Beaverton, OR) within reach, at variable distances from the subjects. The distances depended on the subject's range of motion and the average target for each subject ranged between 12 and 21 cm from the starting position in the Z direction. Approximately 75% of the total area of the monitors was within the robot’s workspace. The subject was comfortably seated with the robot handle positioned in front of their chest. The goal of the task was to guide the robot to position the stylus in the centre of targets that appeared on the monitors.

EMG signals were recorded from the right and left upper trapezius, and processed as described above. The monitor and HapticMaster positions were recorded using the Optotrak, so that positions on the monitors could be transformed into the HapticMaster coordinate system. All signals were recorded simultaneously and processed at 60 Hz, so as to generate a real-time command signal to control the robot. The velocities predicted by the decoders were sent as kinematic control signals to the robot.

#### Protocols

Each experiment began with a set of training reaches in which EMG and kinematic data were collected for training the models. This involved the robot moving automatically along a straight-line trajectory to a set of 18 targets spanning the reachable area of the monitors, appearing twice in random order. The subject held the handle of the robot and was instructed to gently assist the reach, while the EMG from their right upper trapezius was recorded. We chose this method because we wanted control to be intuitive; it was important that the EMG controlling the reaches corresponded as closely as possible to those a subject would naturally make when attempting to reach while interacting with the robot. The approach would also be appropriate for the end users of the interface who would be unable to generate the reaches themselves. The training reaches were generated using a trajectory model that was linear in the kinematics and the target:
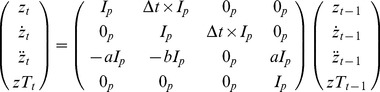
(7)


Here *z_t_* was the stylus position at time *t* and *zT* was the target. The matrix shows the structure for *A* in equation 3, with *I_p_* and *0_p_* being the *p
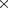
p* identity and zero matrices. The parameters *a* and *b* determined the velocity profile of the trajectory and were both set to 3. Least squares regression was then used to estimate the parameters from the EMG and stylus kinematic data. The state vector consisted of the three-dimensional stylus position, velocity and acceleration. In the case of the KFT parameter estimation, the final recorded position of the stylus, representing the target, was also included.

In testing, the decoders were evaluated with a target acquisition task. A target appeared on one of the monitors one second before an auditory go cue, at which point the subject was free to initiate the reach. The reach was initiated when the RMS feature from either of the EMG channels (left or right upper trapezius) doubled relative to its average value before the go cue. Subjects were instructed to try to perform a natural reach, and if the ipsilateral upper trapezius was activated the reach would initiate. For reaches where the subject would not use the ipsilateral muscle naturally, they could initiate the reach by shrugging their left shoulder. They then guided the robot towards the target with their EMG, through the velocity predicted by the decoder.

Each target consisted of a green circle of 1 cm radius surrounded by 5 rings of various colors, each 1 cm thick. When the movement was complete the color of the target changed to provide performance feedback. For a missed target or if the reach timed out (after 10 seconds) the target turned red, or if one of the rings of the target was attained it turned the color of the corresponding location. For attaining the green circle the subject received a score of 10 points and for outer rings they received 9, 8, 7, 6 and 5 points. Feedback of the cumulative total of their most recent 10 reaches was displayed to them to increase motivation.

The decoder using EMG alone (generic KF) was tested first. Subjects performed 10 practice reaches to become familiar with the decoder, followed by a block of 30 test reaches. Subjects then performed 20 practice reaches of the KFT with perfect target information, to become familiar with the directional model. This was followed either by the mKFT or the mKFT+KF; the order of these models was randomized across subjects. The mixture models were tested in 5 blocks of 32 reaches. For the mKFT, each block included 8 reaches with perfect target information. For the remainder of the reaches, the mixture model was initiated with two potential targets – the correct target and one selected randomly from the workspace. Six reaches each were performed giving the random target a prior of 0.2, 0.5, 0.8 and 1. The order of reaches was randomized within each block. The same format was followed when the generic model was incorporated into the mixture, with a prior probability of *P_G_* = 0.0001. This value was selected as it worked well in some pilot experiments; however, its selection was not optimized. The priors for the remaining targets were reduced by equal amounts as appropriate.

#### Analysis

Reach accuracy was quantified in terms of the target variance accounted for (VAF). While the target VAF was quantified in 3 dimensions for the offline analysis, we used the average of the target VAF in X and Y in the closed-loop case. This made sense as all of the targets were situated on the two monitors resulting in very little variation in the Z dimension. We also calculated the multiple R^2^ between the executed reach and the ideal straight-line reach found using the method for generating training reaches. We evaluated the trajectory straightness by calculating the path efficiencies of the reaches. This was calculated using the ratio of the straight-line distance from the start to the end of the reach to the cumulative distance travelled:
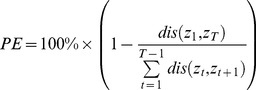
(8)where *dis()* is the distance function. Finally, as for the offline analysis, we looked at the histogram for the weights of the generic model when it was included into the mixture. The decoders were compared using an ANOVA where the combination of *algorithm* and the *prior probability assigned to the random target* was included as a fixed effect, and *subject* was a random effect, and a Tukey post-hoc.

In closed-loop control of the mKFT+KF with no knowledge of the correct target, whether a switch to the generic model occurred may depend on the random target's proximity to the intended target and the difference between the EMG activations for the two trajectories. If the trajectory appeared reasonably accurate to the subject, they may not attempt a switch to the generic model. Furthermore, since the generic model only really provides control in the Y direction in our paradigm (see Results), target differences in X may not be picked up as they may have little effect on the likelihood of the EMG. To investigate these issues we regressed the mean weight assigned to the generic model for each reach on the distance between the random and correct targets in X and Y separately.

## Results

### Offline Evaluation of Natural Reach Decoding with mKFT

**Figure 2:**
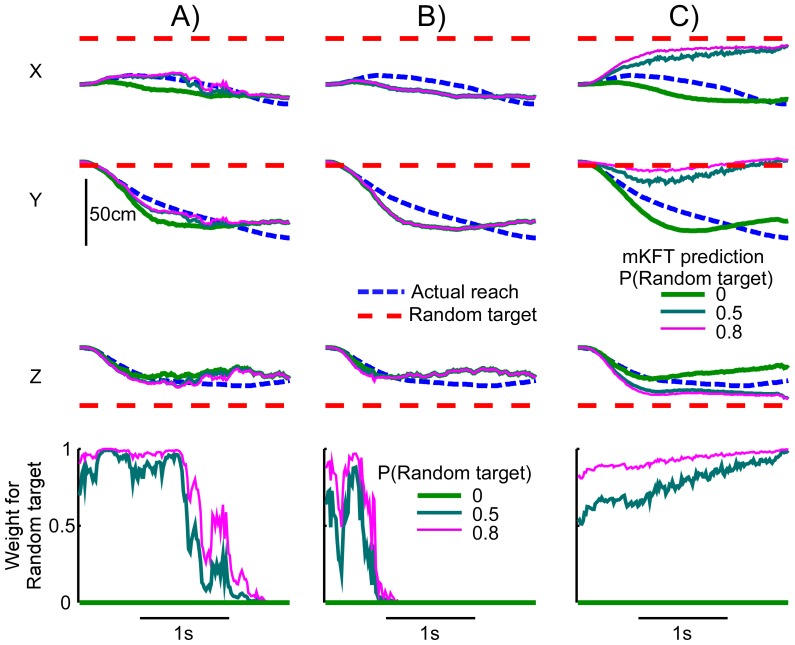
Example reach, mKFT predictions and weights assigned to the random target, with varying target uncertainty at simulated injury levels A) C6; B) C5; and C) C4.

In our offline analysis of natural reaching, we found that the mKFT generally performed well in the face of target uncertainty. However, particularly at the simulated C4 level, the wrong target was sometimes selected. For the example reach below (figure 2), when the random target was given a prior of zero (a prior of 1 for the correct target) the predicted trajectories for the three-dimensional finger position were close to those of the actual reach at all simulated injury levels. At C6 and C5 the decoded reaches were robust to target uncertainty, producing accurate decoding regardless of the prior probability that was assigned to the random target (figure 2 a–b). By examining the weights assigned to each of the trajectories in the mixture, found by multiplying their assigned prior probabilities by the likelihood of the EMG, we can determine which of the trajectories was ultimately selected by the algorithm. At C6 and C5 the weights corresponding to the random target quickly diverged from their assigned priors as the neural data were integrated, and eventually reached zero. For C4 however, there were less neural data and the priors played a larger role in the weighting; when the random target was assigned a prior of 0.5 or 0.8 the decoder made a mistake and selected the wrong trajectory (figure 2c). Such errors rarely occurred for the simulated C6 and C5 conditions, for which more neural data were available.

**Figure 3:**
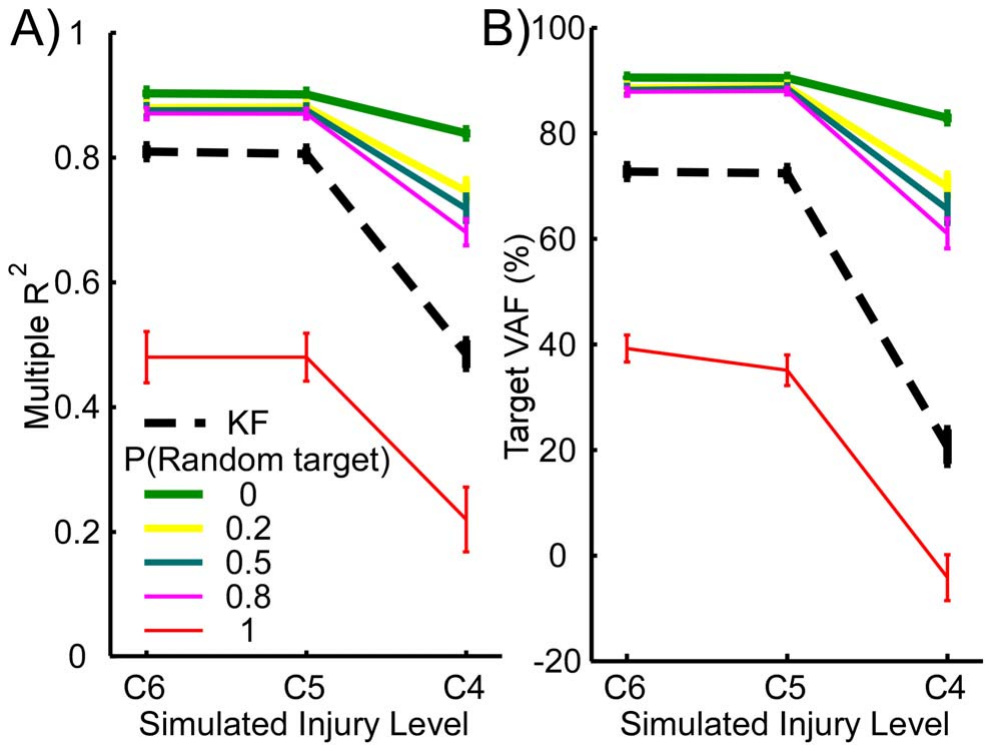
Accuracy with standard errors of A) trajectory and B) target reconstructions for the generic KF, and the mKFT with different prior probabilities assigned to the correct and random targets, with varying the amount of available EMG to simulate different levels of SCI.

Even though the mKFT was less effective at C4 when there was target uncertainty, it was still substantially better than the generic KF with EMG alone, so long as the correct target was represented in the mixture (p<0.001 for all non-zero priors of the correct target, figure 3). The use of the mKFT was clearly worthwhile so long as there was some prior knowledge of the correct target, however uncertain. On the other hand, when the random target was assigned a prior probability of 1 the performance was dramatically worse for all simulated injury levels (p<0.001 in both R^2^ and target VAF). In the case that the target information was incorrect the generic KF would be far preferable to the mKFT model.

### Offline Incorporation of the Generic Model into the mKFT

**Figure 4:**
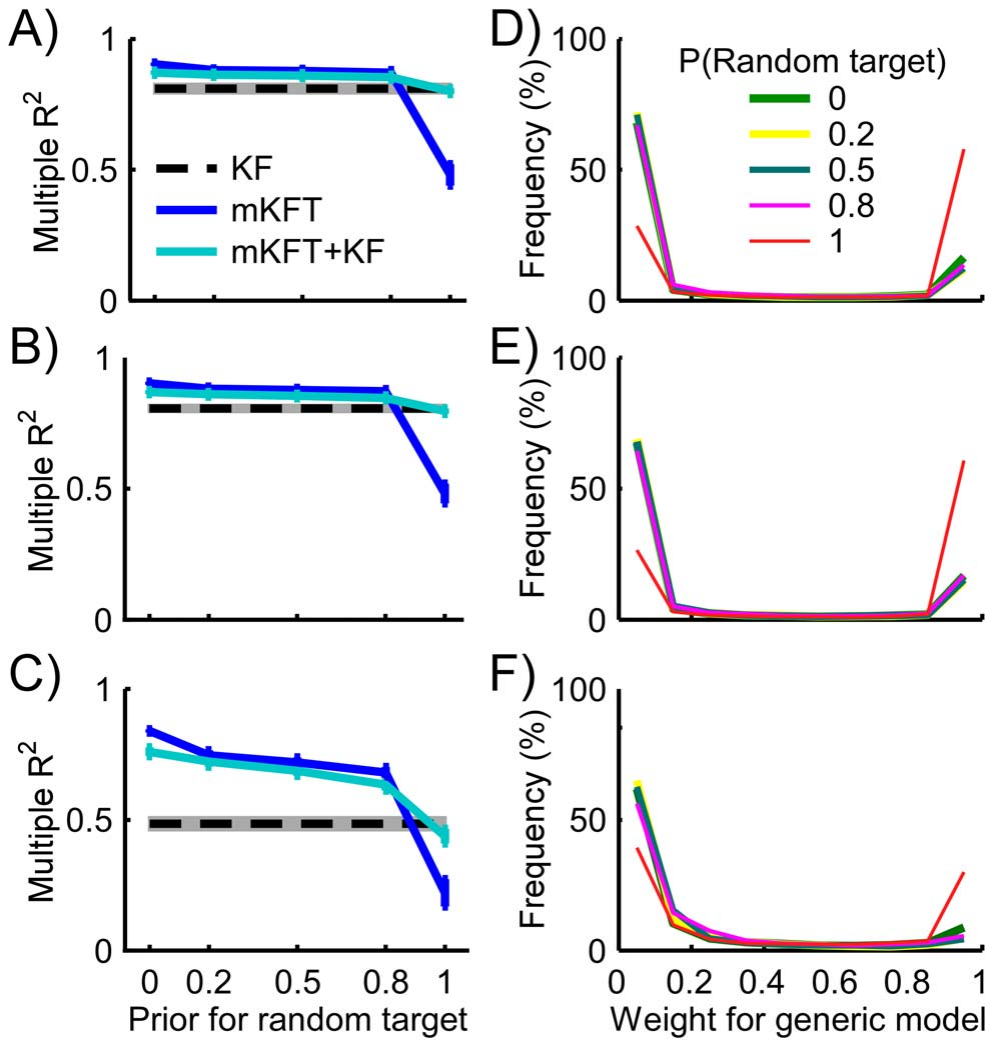
Accuracy of the decoding models with varying target uncertainties for A) C6; B) C5; and C) C4 simulated injury levels. d)-f) Corresponding histograms for the weights assigned to the generic model, over the course of all reaches, in the combined mixture case. The histograms are very similar where the prior assigned to the random target is less than 1. Error bars and shading represent standard errors.

Incorporating the generic model as a mixture component greatly increased performance of the mixture model when the target information provided was wrong (p<0.001, figure 4 a–c). In fact, the accuracy of the mKFT+KF for cases when the random target prior was set to 1 was only slightly lower than that of the generic model alone, and this difference was not statistically significant (p = 0.91). In such cases, while the generic KF was assigned a prior probability of 0.1, its weight quickly increased as the likelihood of the EMG provided evidence that the KFT component was less accurate.

The histograms for the generic model's weight pool data from the entire time-courses of the reaches, and show little difference depending on the prior for the random target, so long as the correct target was included (figure 4 d–f). There was, however, a small cost to the accuracy of the mKFT in these cases, as a higher weight was assigned to the generic KF for a low proportion of the time. This suggests that the predictions from the generic model better described the observed EMG than those from the KFT components. This may occur for less stereotypical movements that did not conform to the structured KFT trajectories, or also in cases when the generic model captured the trajectories particularly well. When the correct target was included, the proportion of the time that the generic model had a zero weight declined as the amount of available EMG was reduced – the generic model was assigned low, non-zero weights for some of the reaches in the C4 case. With so little EMG available, the mixture model may have been less likely to quickly converge to a specific trajectory in some cases. Nonetheless, the generic mixture component did a good job avoiding the dramatic drop in performance when the target information was inaccurate, being selected a higher proportion of the time for all simulated injury levels.

### Closed-loop Evaluation of the Mixture Models

**Figure 5:**
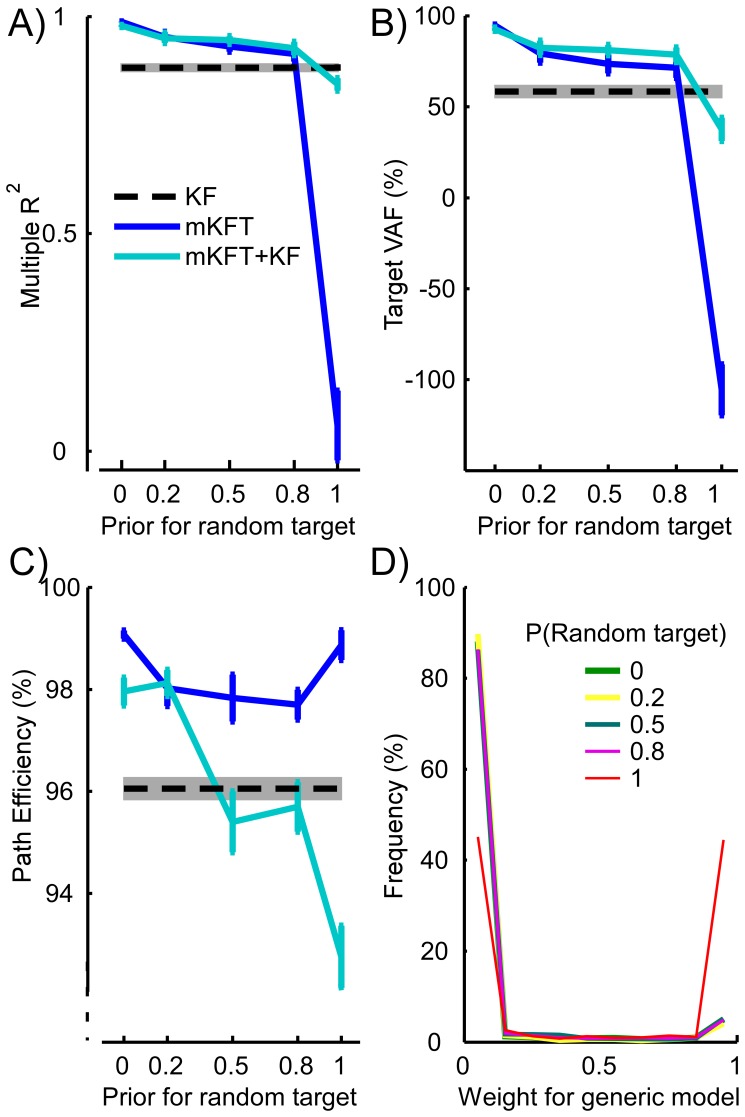
Closed-loop decoder performance with increasing target uncertainty, at the simulated C4 injury level. A) trajectory comparison to straight-line reach; B) target accuracy; C) quantification of the trajectory straightness; D) histograms for the weights assigned to the generic model when it was included. The histograms are almost identical where priors for the random target are less than 1. Error bars and shading represent standard errors.

**Figure 6:**
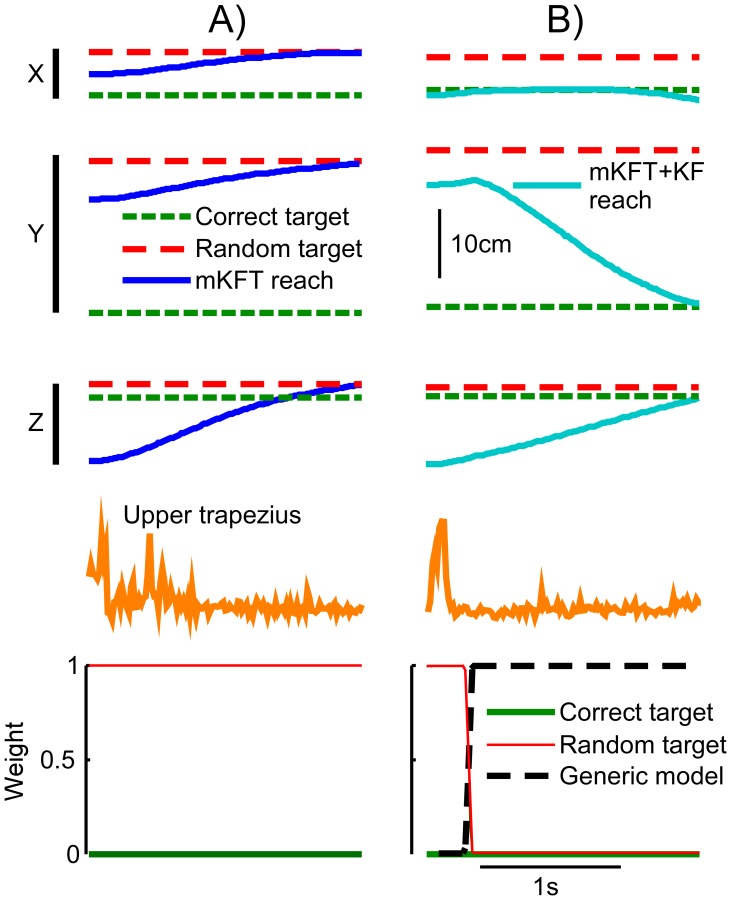
Example reaches with inaccurate target information, where the random target was assigned a prior probability of 1. 3-dimensional robot endpoint position, RMS feature of EMG windows, and mixture weights with A) mKFT and B) mKFT+KF, incorporating the generic model.

In closed-loop control as in the offline analysis, we found that reaches were more accurate with target information than with EMG alone so long as the correct target was represented in the mixture. Unless the random target had a prior of 1, the target VAF for both mixture models was higher than the generic model with EMG alone (all p<0.05, figure 5b). When the random target had a prior of 1, the mKFT was dramatically worse (p<0.001). As can be seen in the example reach, despite the effort evident from the activation of the upper trapezius, subjects could not prevent the robot from moving in the wrong direction (figure 6a). However, in cases when the generic model was included its weight often increased as the likelihood of the EMG for the trajectory to the random target was low, allowing the subject to guide the robot with their EMG alone (figure 6b). This resulted in a far higher target VAF (p<0.001, figure 5b), which while slightly lower, was not statistically different from the generic model (p = 0.33).

The multiple R^2^ demonstrated the same trend as seen in the target VAF, however the R^2^ using the generic model was not significantly different to any of the mixture model conditions (all p>0.08, figure 5a), with two exceptions: the mKFT was significantly more accurate when the random model had a prior of 0 (p = 0.04), and was significantly worse when the random model had a prior of 1 (p<0.001). The primary reason for this is that the R^2^ measures the accuracy of the entire trajectory by, somewhat artificially, comparing it to an “ideal” straight reach. Also, because subjects received feedback and could interact with the decoder, the performance with EMG alone was much better than seen in the offline analysis (compare figure 5a to figure 4c). Specifically, as we found in our previous study, using the upper trapezius alone subjects could control the robot position almost perfectly in the vertical (Y) direction, whereas they had no horizontal (X) control [14]. Because of the constraints of our experimental setup the majority of the workspace variance was in the Y dimension, resulting in higher R^2^s overall and a reduced emphasis on the target accuracy using this measure.

Switching between mixture components naturally resulted in less straight reaches, as evidenced by the path efficiencies (figure 5c). The mKFT without the generic component had some reduction in path efficiency when two targets were in the mixture, indicating that some switching between potential targets occurred. Path efficiencies were not significantly different between the two mixture models when the prior for the random target was 0 or 0.2, but for 0.5 and higher the mixture incorporating the generic component produced significantly lower path efficiencies (all p<0.05). While it was lowest for the worst-case scenario where the random target prior was 1, it was not statistically different from the generic model at priors of 0.5 and 0.8 (both p>0.98). This strongly suggests that the generic model was sometimes selected even when the correct target was in the mixture, resulting in less straight reaches driven by the subjects' EMG alone. We can see that the weight for the generic model was close to 1 for a very small proportion of the time even when the correct model was in the mixture (figure 5d).

**Figure 7:**
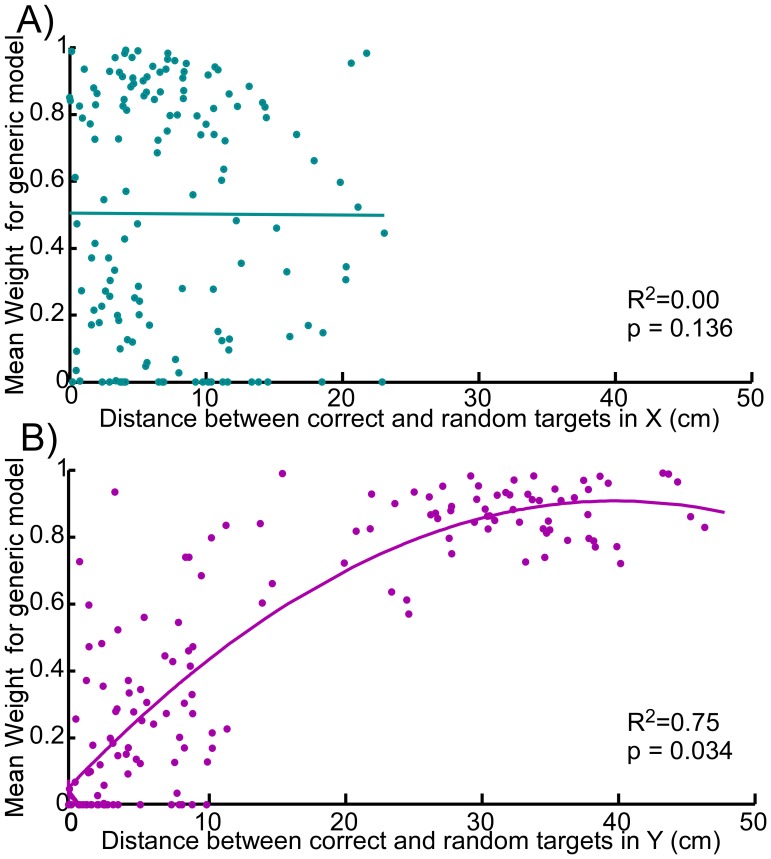
The mean weight assigned to the generic model for each reach where only the random target was in the mixture, plotted against the error between the target location and the random target used by the decoder in A) X with linear fit; and B) Y with quadratic fit. Regression functions are shown with corresponding R^2^ and p statistics.

When the correct target was not in the mixture (i.e. the prior for the random target was ∼1) the mKFT+KF switched to the generic model approximately half of the time (figure 5d). Note that the histogram includes data from the entire time-course of all of the reaches, and the weight was always initially close to zero, so this may under-represent switches that occur later in the reach. Regression analyses showed that the average weight for the generic model was related to the distance between the correct target and the random target used by the decoder in Y (R^2^ = 0.75, p = 0.034, quadratic fit), while it had no relationship to the distance in X (R^2^ = 0, linear fit) (figure 7). As would intuitively be expected given the natural role of the upper trapezius muscle, switches to the generic model were more likely when there was a large discrepancy in the vertical direction. This also suggests that subjects mostly used the generic model to correct for target errors in Y.

## Discussion

The incorporation of target information from gaze or other sources can substantially improve control of a neuroprosthetic reaching interface, particularly when the set of available neural signals is extremely limited. We cannot expect a user's gaze information to be highly informative at all times, however. Here we evaluated target information of varying quality to see how our approach might perform in a more realistic environment where the target information could become unreliable. We found that the mixture model did a good job of selecting the correct target in the face of uncertainty, providing an advantage over a generic model that did not include target information in all cases when the actual target was included in the mixture. We also avoided a large degradation in performance when the target estimate was incorrect by incorporating the generic model into the mixture.

The mixture including the generic model was clearly superior to the mixture without it when the target information was inaccurate; however it was also interesting to compare the performance of the two mixture models when the correct target was included in the mixture. While there were no statistically significant differences between the two models in either the offline analysis or in online control when the correct target was included, the performance without the generic model was consistently slightly better offline, but not in closed-loop. In the offline analysis, the generic model was sometimes selected instead of the correct target, causing a small drop in average accuracy. Similarly in closed loop, when only the correct target was in the mixture, incorporating the generic model may have been slightly detrimental as it was sometimes selected unnecessarily. However, when the random target was included with priors of 0.5 or 0.8, the mixture incorporating the generic model performed slightly better than the original mKFT (figure 5). Even with the correct target in the mixture, the generic model possibly provided subjects with a little added flexibility for error correction, as evidenced also by the lower path efficiencies. While these differences were not significant statistically, it seems that subjects could better utilize the generic model to compensate for errors in closed loop control than occurred in the offline analysis.

Yu et al. previously demonstrated the effectiveness of the mixture model approach with up to 8 potential targets in their mixture of trajectory models paper, decoding with a rich data set of intracortical single neuron recordings [12]. Their approach was slightly different, defining separate trajectory models for a set of pre-defined targets. This would not necessarily allow reaches within a continuous workspace as was demonstrated here, as fast switching between the models would make it difficult to balance the weights to achieve intermediate targets. However, even when the eight targets were initialized with uniform priors the decoding was very effective. Given those results it was not surprising that our mixture model worked well with uncertainty when there was sufficient EMG available. What is interesting is how well the model performed with just a single EMG channel, particularly in closed-loop control. The fact that subjects could easily learn and adapt to this non-stationary interface was somewhat surprising. Evidently, there was a substantial amount of switching between models when the target information was more uncertain and the generic model was included. Even so, subjects did not report a noticeable difference in difficulty between the two mixture models and they were able to take advantage of the generic model when it was available to improve accuracy.

The subjects' apparent comfort with switching between trajectory models is encouraging, and suggests that further extensions to this approach may be feasible. The interface in its current form allows only for discrete reaches where potential targets are identified in the period preceding reach initiation, while the gaze data during control of the reaches are unused. An interesting avenue for future research would be to look at monitoring the gaze continuously during reach control and allowing new mixture components to be introduced, particularly if the neural data is not a good fit to the active trajectory. Researchers have also developed decoders to identify state transitions, such as between posture and movement classes to identify reach initiation [19], [20]. Such higher-level approaches may also be useful in developing a more continuous, gaze-dependent reach control, which could potentially allow users to correct more accurately for errors than in the current work, or even to change their minds about the target mid-reach.

The main advantage to incorporating gaze that we have found in our work has been allowing accurate reaching while reducing the burden on the user [14]. Adding further signal sources has the potential to complicate a control interface, but we found that using the target estimates to enhance the trajectory model created an intuitive mapping. It is possible that the addition of the generic model may increase the complexity as users must avoid activating that model when moving along an accurate trajectory. If things were going wrong the ability to switch to entirely neural control would certainly be extremely valuable, but whether this comes at a cost of additional burden to the user is unknown. One way to deal with this might be to select the prior probability for the generic model for an optimal trade-off between accidental switches to the generic model and ease of switching in the case of inaccurate target information, an issue that we have not addressed here. Reassuringly, we found in this study that unwanted switches to the generic model were infrequent, and subjects did not report finding the mixture incorporating the generic model to be more challenging than the standard mKFT. We hope that by allowing neuroprosthesis users to take control with their neural signals they will be able to more safely use target-dependent interfaces that enable more intuitive ease of control.
